# Traumatic Cervical Spine Injury in Children and Adolescents Requiring Neurosurgical Intensive Care: A Seven-Year Retrospective Descriptive Cohort

**DOI:** 10.3390/neurosci7050110

**Published:** 2026-10-06

**Authors:** Vikas Chauhan, Gyaninder Pal Singh

**Affiliations:** 1 Department of Anesthesiology, University of Mississippi Medical Center, 2500 North State Street, Jackson, MS 39216, USA; 2 Department of Neuroanaesthesiology and Neurocritical Care, All India Institute of Medical Sciences, New Delhi 110029, India

**Keywords:** traumatic cervical spine injury, children and adolescents, neurosurgical intensive care, ASIA Impairment Scale, retrospective cohort, discharge neurological status

## Abstract

Traumatic cervical spine injury in children and adolescents is uncommon but may cause major disability and prolonged critical care. This retrospective descriptive cohort characterizes patients aged 18 years or younger admitted to the Neurosurgical Intensive Care Unit (ICU) of a tertiary trauma center in New Delhi, India, during 2008–2015. Discharge status was classified as favorable (American Spinal Injury Association [ASIA] Impairment Scale D–E) or unfavorable (ASIA A–C or in-hospital death). We used chi-square tests with Yates’ continuity correction, with unadjusted odds ratios (ORs) and 95% confidence intervals (CIs). Among 112 patients, 83.0% were male, 85.7% were aged 9–18 years, and 77.7% had spinal canal compromise. Forty-seven patients (42.0%) had favorable and 65 (58.0%) had unfavorable discharge status, including nine in-hospital deaths (8.0%). Favorable status was associated with absence of spinal canal compromise (OR 6.67, 95% CI 2.40–18.54) and absence of the composite ICU complication indicator (OR 6.44, 95% CI 2.61–15.93), and with fracture rather than subluxation, absence of tracheostomy, and favorable admission ASIA category. No multivariable adjustment was performed; these exploratory, unadjusted associations do not establish independent or causal effects. The cohort provides descriptive data from an under-reported tertiary trauma setting and identifies variables for prospective multicenter study.

## 1. Introduction

Traumatic cervical spine injury in children and adolescents is uncommon but carries a disproportionate risk of neurological disability, mortality, prolonged hospitalization, and resource-intensive multidisciplinary care [1,2,3,4,5]. Global Burden of Disease estimates indicate that the burden of pediatric spinal cord injury is highest among males and adolescents aged 15–19 years, with falls as the leading cause [6]. Mechanisms vary by age and setting, with road traffic trauma, falls, sports-related injury, and non-accidental trauma contributing in different proportions [1,2,7,8]. Developmental anatomy, including a relatively large head, ligamentous laxity, shallow facet joints, incomplete ossification, and greater cervical mobility, also produces age-dependent injury patterns that differ from those in adults [9,10,11].

Severe cervical spine injuries may require operative stabilization and admission to intensive care for airway protection, hemodynamic support, ventilation, and prevention of secondary neurological injury [12,13,14,15,16,17]. The ASIA Impairment Scale is commonly used to grade neurological severity, and admission grade is associated with subsequent neurological status [18,19,20]. Catastrophic dislocation may produce profound neurological injury [21], while radiological features such as spinal canal compromise and unstable injury patterns may further indicate anatomical severity [11,13,14].

Airway management and intensive care are clinically important components of treatment, but post-admission interventions and complications are closely intertwined with baseline injury severity and duration of critical illness [15,16,17,22,23]. Most available series emphasize epidemiology, imaging, or surgical management, whereas integrated descriptions of high-acuity cohorts requiring neurosurgical intensive care remain comparatively limited, particularly outside large registries and multicenter systems [3,4,24,25,26,27]. Pediatric data from South Asia are comparatively sparse, although modeling suggests that the World Health Organization South-East Asia Region, which includes India, carries the largest projected number of pediatric traumatic spine injuries worldwide [28]. Single-center pediatric series from India and Pakistan [29,30,31,32,33] were not restricted to patients requiring neurosurgical intensive care, and data on the critical care burden of cervical spinal cord injury, including respiratory morbidity and ventilator dependence, have been reported from an Indian level I trauma center in adults [34]. Historical cohorts must also be interpreted in light of evolving imaging, airway devices, operative techniques, and critical care practice.

The primary objective of this study was to describe the demographic characteristics, injury patterns, management features, ICU course, and discharge outcomes of children and adolescents with traumatic cervical spine injury admitted to a tertiary neurosurgical ICU. A secondary exploratory objective was to evaluate unadjusted associations between selected clinical variables and discharge neurological status.

## 2. Materials and Methods

### 2.1. Study Design, Setting, Participants, and Ethics

This retrospective descriptive cohort study was conducted in the Neurosurgical Intensive Care Unit (ICU) of a tertiary-level trauma center at the All India Institute of Medical Sciences, New Delhi, India. Medical records from the seven-year study period (2008–2015) were reviewed after approval by the Institutional Ethics Committee of the All India Institute of Medical Sciences, New Delhi (IEC-122/05.02.2016). The requirement for individual informed consent was waived because the study involved retrospective review of anonymized clinical records. Inclusion criteria were age 18 years or younger, traumatic cervical spine injury, and admission to the Neurosurgical ICU during the study period. Non-traumatic cervical spine disorders and patients older than 18 years were not eligible. The present analysis includes 112 eligible patients. The number of records screened for eligibility is not available. Because the cohort includes older adolescents, the population is described throughout as children and adolescents.

### 2.2. Data Sources and Variables

Variables included in the present analysis were age group (0–8 or 9–18 years), sex, mechanism of injury (fall or road traffic accident), injury type (fracture, subluxation, or spinal cord injury without radiographic abnormality [SCIWORA]), radiological spinal canal compromise, and admission ASIA category. Management variables were operative versus non-operative treatment, intubation technique among operative cases, tracheostomy, and a composite indicator of any documented ICU-related or hospital-acquired complication.

The ICU complication variable was analyzed as a composite present/absent indicator and was not disaggregated into individual complication categories such as respiratory, urinary, or bloodstream infection. We analyzed operative treatment as a binary operative-versus-non-operative variable and did not separate it into stabilization, decompression, or combined procedures; procedure-specific categories such as anterior fusion, posterior fusion, closed reduction, and decompressive laminectomy, and the spinal levels addressed, are not available for this report. Intubation technique was classified as direct laryngoscopy, fiberoptic bronchoscopy, or video laryngoscopy among the 101 patients who underwent operative management; technique selection was at the discretion of the attending anesthesiologist, and the reasons for individual choices are not available. Narrower age strata within the 9–18-year category, body weight, injured spinal levels, multilevel involvement, associated injuries, and intraoperative hemodynamic variables were not included in the present analysis. This descriptive report is limited to the aggregate categories reported in the original study tables; no additional subgroup categories were imputed or reconstructed. The three-category discharge distribution in Table 2 and the bounds described for Table 4 were derived arithmetically from the reported counts. The conservative management of the single patient with SCIWORA was reported by the study investigators and is not derived from the aggregate tabulations.

### 2.3. Outcome Measure

The primary outcome was neurological status at hospital discharge. A favorable status was defined as ASIA D or E; an unfavorable status was defined as ASIA A, B, or C, or in-hospital death. Admission ASIA category was dichotomized as favorable (D–E) or unfavorable (A–C), and this binary classification was retained from the original study design. Because in-hospital death is included only in the unfavorable discharge category, movement from the favorable admission category to the unfavorable discharge category cannot be interpreted solely as neurological deterioration.

### 2.4. Statistical Analysis

Categorical variables are summarized as numbers and percentages. Between-group comparisons used the chi-square test with Yates’ continuity correction for 2 × 2 tables. Unadjusted odds ratios (ORs) with 95% confidence intervals (CIs) were calculated from the corresponding contingency-table counts. The single SCIWORA case was excluded from the fracture-versus-subluxation comparison. All reported aggregate 2 × 2 analyses were independently reproduced and verified by the authors using jamovi version 28.2 (The jamovi project, Sydney, Australia). All tests were two-sided, and *p* < 0.05 was considered statistically significant. No correction was made for multiple comparisons. The analyses are exploratory and unadjusted. No multivariable model was fitted, and the estimates do not establish independent prognostic effects.

### 2.5. Use of Generative Artificial Intelligence

Generative artificial intelligence tools (ChatGPT version 5.5, OpenAI; Claude version 5.0, Anthropic) were used during manuscript preparation for language editing, drafting, and manuscript organization. ChatGPT was also used during preliminary verification of the aggregate statistical calculations; all analyses reported in Section 2.4 were subsequently independently reproduced and verified by the authors in jamovi version 28.2 (The jamovi project, Sydney, Australia). The authors reviewed all AI-assisted output, verified the cited sources and numerical statements, and take responsibility for the final analysis and manuscript.

## 3. Results

A total of 112 children and adolescents with traumatic cervical spine injury were included. Ninety-three patients (83.0%) were male, and 96 (85.7%) were aged 9–18 years. Falls accounted for 66 injuries (58.9%) and road traffic accidents for 46 (41.1%). Fractures were present in 68 patients (60.7%), subluxations in 43 (38.4%), and SCIWORA in one (0.9%). Spinal canal compromise was documented in 87 patients (77.7%). At admission, 68 patients (60.7%) had an unfavorable ASIA category (A–C), and 44 (39.3%) had a favorable category (D–E) (Table 1).

Operative management was recorded in 101 patients (90.2%), while 11 (9.8%) were managed non-operatively. Among operative cases, direct laryngoscopy was used in 74 (73.3%), fiberoptic bronchoscopy in 25 (24.8%), and video laryngoscopy in two (2.0%). Tracheostomy was performed in 34 patients (30.4%), and the composite ICU complication variable was present in 45 (40.2%). At hospital discharge, 47 patients (42.0%) had a favorable neurological status and 65 (58.0%) had an unfavorable status, including nine in-hospital deaths (8.0%); accordingly, 56 patients (50.0%) survived to discharge with ASIA A–C (Table 2).

In exploratory unadjusted analyses, favorable discharge status was associated with absence of spinal canal compromise (OR 6.67, 95% CI 2.40–18.54; *p* < 0.001), absence of the composite ICU complication indicator (OR 6.44, 95% CI 2.61–15.93; *p* < 0.001), fracture rather than subluxation (OR 2.74, 95% CI 1.21–6.21; *p* = 0.024), absence of tracheostomy (OR 2.64, 95% CI 1.09–6.37; *p* = 0.047), and favorable admission ASIA category (OR 2.35, 95% CI 1.08–5.11; *p* = 0.048). Age group, sex, and mechanism of injury were not significantly associated with discharge status. The single SCIWORA case was managed conservatively, without surgery, and had an unfavorable outcome; the aggregate data do not distinguish ASIA A–C at discharge from in-hospital death. These marginal associations do not account for baseline severity or other confounding variables (Table 3).

The dichotomized admission-to-discharge cross-tabulation is shown in Table 4. Of the 44 patients in the favorable admission category, 24 remained in the favorable category at discharge and 20 crossed into the unfavorable category. Of the 68 patients in the unfavorable admission category, 23 crossed into the favorable category at discharge, and 45 remained in the unfavorable category. Because the favorable discharge category excludes death, all 23 patients who moved from admission A–C to discharge D–E were alive at discharge and improved from the unfavorable to the favorable category. Because the cohort included nine in-hospital deaths in total, at least 11 of the 20 patients who moved from admission D–E to an unfavorable discharge status survived with ASIA A–C at discharge. Individual A–E grades were not reported in the original aggregate tables, so the magnitude of grade change within these movements cannot be described.

## 4. Discussion

### 4.1. Principal Findings

This retrospective cohort describes 112 children and adolescents with traumatic cervical spine injury requiring neurosurgical intensive care over a seven-year period. The cohort was predominantly male and aged 9–18 years; falls were the most common mechanism; spinal canal compromise was frequent; and 90.2% underwent operative management. At discharge, 58.0% had an unfavorable neurological status, including an in-hospital mortality of 8.0%. Selected anatomical, clinical, and ICU-course variables were associated with discharge status in unadjusted analyses. The principal contribution of the study is descriptive characterization of a selected high-acuity cohort rather than development of an independent prognostic model.

### 4.2. Demographic and Mechanistic Profile

The male predominance (83.0%) and concentration of patients in the 9–18-year group (85.7%) are broadly consistent with reports showing increasing exposure to high-energy mechanisms among older children and adolescents [3,4,24,25]. Falls accounted for 58.9% of injuries and road traffic accidents for 41.1%, a distribution that differs from settings in which motor vehicle trauma predominates [1,2,24,26]. Favorable discharge status occurred in 39.4% of patients injured by falls and 45.7% after road traffic accidents; this difference was not statistically significant. The broad age category and the small number of patients aged 0–8 years prevent reliable developmental subgroup comparisons, and the cohort should therefore be interpreted as one of children and adolescents rather than younger children alone.

### 4.3. Comparison with Indian and Neighboring-Country Series

Selected demographic features resemble those of other Indian series, although case mix differs among them. In 84 children and adolescents with cervical spine injury treated at a South Indian neurosurgical institute, 79% were aged 13–18 years, the mean age was 14.7 years, and boys outnumbered girls by approximately six to one [29]. In a North Indian series of 75 pediatric cervical spine injuries, 84% of patients were aged 10–18 years and 80% were boys [31]. A review of 204 pediatric spinal injuries at all levels from a dedicated Indian spinal injuries center reported a mean age of 15.69 years and a threefold male excess [30]. The present cohort, in which 85.7% of patients were aged 9–18 years and 83.0% were male, shows a similar adolescent and male predominance. The age bands differ among these studies, however, and none was restricted to patients admitted to a neurosurgical ICU, so the similarity concerns demographic pattern rather than case mix.

The rank order of mechanisms is also similar. In the North Indian series, fall from height accounted for 44% of injuries and road traffic accidents for 36%, with the remainder attributed to other mechanisms [31]; in the present cohort, in which mechanism was recorded in only two categories, falls accounted for 58.9% and road traffic accidents for 41.1%. Falls have been reported as the predominant mechanism in Indian and other developing-country series, in contrast to the motor-vehicle predominance reported from many high-income settings [30,31]; in a meta-analysis of pediatric traumatic spinal cord injury, falls were the leading cause in developing countries (31%, from five studies) and transport injuries in developed countries (50%) [35]. Outcome comparisons require matched endpoints. In the North Indian series, 11 of 75 patients (14.7%) died during hospitalization, and a further four died after discharge, giving the reported overall mortality of 20% [31]; the in-hospital figure is therefore the closer comparator for the 8.0% in-hospital mortality observed here. Even this comparison is imperfect: 17% of patients in that series left hospital against medical advice, which limits the ascertainment of in-hospital outcomes [31]; discharge against medical advice is not available as a separate category in the present data; and the present cohort comprises only patients admitted to a neurosurgical ICU. Surgical management was reported in 40% of the North Indian series, predominantly through anterior approaches [31], compared with 90.2% here, a difference that may partly reflect the selection of the present cohort by neurosurgical ICU admission, as well as differences in injury severity, referral patterns, and surgical practice.

Pediatric data from other neighboring countries are sparse: a scoping review of pediatric traumatic spinal cord injury in low- and middle-income countries identified only five studies reporting management or outcomes [36]. The closest comparator is a prospective series of 187 children aged 6–17 years with cervical spine injury treated at a tertiary hospital in Peshawar, Pakistan, during 2021–2022 [33]. Falls and motor vehicle accidents were the leading mechanisms, the upper cervical spine (C0–C2) was involved in 54% of patients, 87.2% were graded ASIA A–C at presentation (compared with 60.7% at admission here), and 143 (76.5%) underwent surgery, alone or combined with traction or immobilization [33]. Mortality in that series was 27.8% during a mean follow-up of 3.8 months, and in-hospital deaths were not reported separately, so it cannot be compared directly with the 8.0% in-hospital mortality reported here; the cohorts also differ in study period and age range. In a rehabilitation-center series of 102 children up to 15 years of age with spinal cord injury at all levels from the same city, firearm injury was the most common mechanism (38.2%), bomb blast accounted for 6.9%, and only 13.7% of injuries were cervical [32], illustrating how strongly mechanism and case mix vary within the region. In Kathmandu, Nepal, spinal injuries accounted for 4% of 352 children admitted with acute neurotrauma during 2001–2004, and children with spinal injuries sustained outside the Kathmandu Valley took more than eight days to reach neurosurgical care [37]. For China, Global Burden of Disease modeling shows that the incidence of pediatric spinal cord injury declined between 1990 and 2021, in parallel with the global decline, but projects an increase by 2045 [6]; these are modeled population estimates for spinal cord injury at all levels rather than clinical cohort data. Section 4.5 considers clinical data from China on SCIWORA.

### 4.4. Comparison with Series from High-Income Settings

National registry analyses from the United States provide numerical comparators, although selection and definitions differ. Among 2538 children aged 17 years or younger with cervical spinal cord injury in the Trauma Quality Improvement Program (2017–2021), motor vehicle collision was the most common mechanism (37.8%), falls accounted for 26.1%, and mortality was 13% [38]. In a National Trauma Data Bank analysis of 4696 patients aged 18 years or younger with cervical spinal cord injury (2007–2018), adolescents accounted for 71.9% and children for 20.5% of cases; the highest mortality occurred among newborns and infants (25.6%) and toddlers (22.1%); motor vehicle trauma predominated in younger children; and firearm injury was more common among adolescents [39]. Those age strata are not directly comparable with the single 9–18-year category used here, and with only 16 patients aged 0–8 years the present cohort cannot examine an age gradient in mortality. The contrast in mechanism is nonetheless evident: falls accounted for more than half of the injuries in the present cohort, compared with about a quarter in the U.S. registry data, and penetrating mechanisms, which contribute to the U.S. data, were not among the mechanisms recorded here. The registry cohorts include children admitted to participating trauma centers regardless of ICU admission, whereas the present cohort was restricted to neurosurgical ICU admissions; this difference in selection, together with differences in outcome ascertainment, precludes direct comparison of mortality. Series from Germany, Portugal, Australia, and Saudi Arabia describe injury patterns and intensive care needs in other health systems [3,4,24,25].

### 4.5. Injury Pattern, Canal Compromise, and SCIWORA

Spinal canal compromise was present in 77.7% of patients, and 67.8% of those patients had an unfavorable discharge status. Absence of canal compromise was associated with higher odds of favorable status, while fracture injuries were associated with more favorable status than subluxations. These findings are directionally consistent with literature linking canal narrowing and unstable injury patterns to neurological severity [13,26,40,41,42]. However, canal compromise, injury pattern, and admission ASIA category are clinically interrelated; the unadjusted estimates cannot determine which feature has an independent effect. The single SCIWORA case also precludes inference regarding that subgroup. Early imaging remains essential for diagnosis and management, but the present data should not be used as an independent radiological prediction model [9,10,11,13].

SCIWORA was identified in only one patient (0.9%). Considerably higher proportions have been reported elsewhere, particularly among younger children: in the North Indian series, SCIWORA occurred in 42% of children aged 0–9 years and 8% of those aged 10–18 years [31]; SCIWORA has been reported to account for 13–42% of pediatric spinal cord injuries [43] and for 35% of pediatric traumatic spinal cord injuries in a meta-analysis of studies from developed countries, which found no corresponding data from developing countries [35]. The 13–42% range and the pooled 35% are calculated among children with spinal cord injury, whereas the denominator here includes all cervical spine injuries, with or without cord involvement, so the figures are not directly comparable. Several explanations are possible, none of which can be tested with these data. SCIWORA occurs predominantly in younger children [31,43], and only 14.3% of this cohort was aged 0–8 years. Its detection depends on magnetic resonance imaging [3,10], and imaging practice during the study period cannot be assessed here. SCIWORA may also involve the thoracic rather than the cervical cord, as in 77% of a Chinese series of 47 children, most of whom were younger than eight years and were injured during sports, particularly dance backbend practice [43]; such cases would not enter a cervical cohort, and children with cervical SCIWORA who did not require intensive care may likewise not have been admitted to the neurosurgical ICU. In that Chinese series, 98% of children with SCIWORA were managed conservatively [43], as was the single patient with SCIWORA in the present cohort. Further details of that patient’s management are not available in the data underlying this report, and one case does not support inference about this subgroup.

### 4.6. Operative Management and Airway Technique

Operative management was recorded in 90.2% of the cohort, reflecting the selected high-acuity population admitted to a neurosurgical ICU. Specific operative procedures were not available in the aggregate study categories, so no distinction can be made among stabilization, decompression, or combined approaches. Direct laryngoscopy was used in 73.3% of operative cases, fiberoptic bronchoscopy in 24.8%, and video laryngoscopy in 2.0%. These frequencies describe practice during 2008–2015 and should not be interpreted as evidence that one airway technique was superior or as a recommendation for current practice. Cervical immobilization, careful airway planning, and avoidance of secondary injury remain central principles [15,16,17,22,23,44].

The low proportion of video laryngoscopy warrants comment. During the study period, the choice of intubation technique, including whether to use video laryngoscopy, was at the discretion of the attending anesthesiologist for each patient. The factors that informed individual decisions, such as device availability, operator experience, the urgency of intubation, and anticipated airway difficulty, are not available in the data underlying this report, so the reasons for the observed distribution cannot be determined from this cohort. Two external reference points provide context without explaining the local pattern. A national survey of members of the Indian Society of Neuroanaesthesiology and Critical Care, conducted after the study period, found that respondents preferred indirect techniques for elective intubation of patients with cervical spine instability but direct laryngoscopy for emergency intubation [44]; because the urgency of intubation was not available here, the relevance of that pattern to this cohort is uncertain. More recently, multi-society guidelines have recommended videolaryngoscopy, where possible, for tracheal intubation in patients with suspected or confirmed cervical spine injury, noting that awake tracheal intubation had historically been regarded by many as the gold standard and that the supporting evidence is generally modest [45]. Those guidelines were developed for adults and are not pediatric-specific. They also found no data suggesting that direct laryngoscopy increases the risk of secondary spinal cord injury [45], so the predominance of direct laryngoscopy in this cohort should not be read as a safety signal.

### 4.7. Intensive Care Course, Tracheostomy, and Complications

The composite ICU complication variable was present in 40.2% of patients, and tracheostomy was performed in 30.4%; both were associated with unfavorable discharge status in unadjusted analyses. Both variables were recorded after admission and were influenced by baseline neurological severity, respiratory failure, duration of ventilation, and time at risk in the ICU. Tracheostomy is additionally susceptible to confounding by indication, whereas the complication composite is susceptible to severity-related and time-dependent confounding. In patients with traumatic brain injury, for example, the need for tracheostomy has been associated with markers of injury severity such as the Glasgow Coma Scale score [46]; whether analogous severity markers account for the association observed here cannot be assessed. Consequently, neither association can be interpreted causally. They are better regarded as markers of a severe or complicated clinical course. In adults with cervical spinal cord injury treated at the All India Institute of Medical Sciences, New Delhi, during 2017–2018, poor ASIA grade and prolonged ventilator dependence, ICU stay, and hospital stay were associated with mortality [34]; the tracheostomy proportion in the present pediatric cohort is consistent with a substantial respiratory burden in this setting, although age, study period, and case mix differ. The composite complication measure also combines events of differing type, severity, and timing. Prevention of secondary insults remains a core objective of spinal cord critical care [13,47,48,49], but the present cohort cannot quantify the effect of any specific ICU intervention.

### 4.8. Neurological Status at Admission and Discharge

At admission, 60.7% of patients were in the unfavorable ASIA category (A–C), and a favorable admission category was associated with favorable discharge status. This is consistent with the recognized prognostic relevance of the ASIA Impairment Scale [12,18,19,20]. The dichotomized cross-tabulation in Table 4 shows movement in both directions between the admission and discharge categories, and the composite outcome affects the two directions differently. The 23 patients who moved from admission A–C to discharge D–E were alive at discharge, so this cell represents improvement in the recorded dichotomized category; it does not indicate the magnitude of grade change, functional recovery, or a treatment effect, and the timing of the admission examination was not available. In the opposite direction, in-hospital death limits interpretation of the 20 patients who moved from admission D–E to an unfavorable discharge status but cannot account for all of them: because the cohort included nine deaths in total, at least 11 of these 20 patients survived to discharge with ASIA A–C, a decline in the recorded dichotomized category. Overall, 47 patients (42.0%) were discharged alive with ASIA D–E, 56 (50.0%) were discharged alive with ASIA A–C, and nine (8.0%) died in hospital (Table 2). Individual A–E grades were not available, so grade-level changes within each category cannot be described, and long-term function was not assessed. The findings therefore support category-level description rather than individual-level prediction.

### 4.9. Interpretation in Relation to the Study Period

The cohort was assembled between 2008 and 2015, and several aspects of currently recommended care may differ from the care delivered then. Contemporary series regard magnetic resonance imaging as essential for detecting cord injury without radiographic abnormality [3,10]; current guidelines recommend videolaryngoscopy, where possible, for tracheal intubation in adults with suspected or confirmed cervical spine injury [45]; and current reviews emphasize early decompression and perfusion-directed hemodynamic management within structured neurocritical care [13,47,48,49]. The extent to which care during the study period differed from these recommendations cannot be assessed from the available data. The findings should therefore be read as a description of institutional practice and outcomes during 2008–2015 and not as an audit of current management; because outcomes were not analyzed across time periods, they also provide no evidence of change in prognosis over time. The study was conducted at the All India Institute of Medical Sciences, New Delhi, and the discussion is intentionally limited to this single-center cohort rather than extrapolating to the broader Indian health system. Within that scope, the series adds data from a setting that remains under-represented in many multicenter reports [3,4,24,25,26,27].

### 4.10. Strengths and Limitations

Strengths include the size of the cohort for a rare condition, the seven-year observation period, and integrated reporting of injury, management, ICU-course, and discharge variables. Several limitations must be emphasized. The design is retrospective and single-center, which limits external validity and precludes causal inference, and a cohort selected by neurosurgical ICU admission does not represent all children and adolescents with cervical spine trauma.

The absence of procedure-level surgical data is a major limitation for neurosurgical interpretation. Operative management could not be classified as anterior fusion, posterior fusion, closed reduction, or decompressive laminectomy, or by the spinal levels and approach involved, although these procedures differ in indication, in the segments addressed, and in their perioperative and intensive care implications, particularly between upper and lower cervical injuries [11]. Reported practice also varies within the region: anterior approaches predominated among surgically treated children in a North Indian series, and posterior approaches in a Pakistani series [31,33]. The operative proportion reported here should therefore be read as an indicator of cohort acuity rather than as a description of surgical practice.

The data were collected during 2008–2015, and aspects of care may have changed since then (Section 4.9). Age categories were broad and predominantly represented the 9–18-year group; narrower age strata and body-weight stratification were not included in the present analysis. ASIA grades were dichotomized; in-hospital death was included in the unfavorable discharge composite; individual A–E transition information was not available; and outcome was assessed only at discharge without long-term neurological or functional follow-up. The ICU complication variable is a composite. Injured levels, multilevel involvement, associated injuries, and intraoperative hemodynamic variables were not included. No multivariable adjustment was performed, so residual confounding by baseline injury severity is expected; tracheostomy is susceptible to confounding by indication, whereas ICU complications are subject to temporal ambiguity, severity-related confounding, and differential time at risk. No correction was made for multiple comparisons. The associations are therefore descriptive and hypothesis-generating rather than prognostic. Prospective multicenter studies should use standardized age strata, injury definitions, procedure categories, perioperative physiological variables, complication definitions, and longitudinal neurological outcomes.

## 5. Conclusions

This single-center cohort from 2008 to 2015 describes the characteristics, management, and discharge outcomes of 112 children and adolescents with traumatic cervical spine injury requiring neurosurgical intensive care. Spinal canal compromise, injury type, admission ASIA category, tracheostomy, and the composite ICU complication variable were associated with discharge neurological status in exploratory unadjusted analyses, and a dichotomized admission-to-discharge cross-tabulation is reported. Because no multivariable adjustment was performed, these associations do not represent independent or causal effects, and the post-admission variables may reflect baseline injury severity and the subsequent clinical course rather than modifiable influences on recovery.

Within these constraints, three cautious observations can be offered. First, the adolescent, male, and fall-predominant profile of this cohort resembles that reported in other Indian series and differs from the motor-vehicle-predominant pattern in U.S. registry data, a pattern relevant to injury-prevention planning in this setting. Second, with spinal canal compromise in more than three-quarters of patients and an operative proportion above 90%, children reaching neurosurgical intensive care in this setting formed a high-acuity subgroup whose outcomes should not be generalized to pediatric cervical spine trauma as a whole. Third, the associations of tracheostomy and ICU complications with discharge status are more plausibly interpreted as markers of injury severity and a complicated clinical course than as evidence of modifiable causal pathways.

The principal value of this report is the descriptive characterization of a relatively large, under-reported high-acuity cohort. Prospective multicenter studies with standardized patient-level variables and longitudinal follow-up are required before independent prognostic factors or modifiable care processes can be identified.

## Figures and Tables

**Table 1:** Cohort Demographic and Injury Characteristics.

Characteristic	Category	Number (*n*)	Percentage (%)
Total Patients		112	100.0
Age Distribution	0–8 years	16	14.3
	9–18 years	96	85.7
Sex Distribution	Male	93	83.0
	Female	19	17.0
Age and Sex	0–8 years: Male	10	8.9
	0–8 years: Female	6	5.4
	9–18 years: Male	83	74.1
	9–18 years: Female	13	11.6
Mechanism of Injury	Fall	66	58.9
	Road Traffic Accident	46	41.1
Type of Spinal Injury	Fracture	68	60.7
	Subluxation	43	38.4
	SCIWORA	1	0.9
Spinal Canal Compromise	Present	87	77.7
	Absent	25	22.3
ASIA Category at Admission	Unfavorable (A–C)	68	60.7
	Favorable (D–E)	44	39.3

SCIWORA = spinal cord injury without radiographic abnormality.

**Table 2:** Management and In-Hospital Outcome Characteristics.

Management or Outcome Parameter	Category	Number (*n*)	Percentage (%)
Operative Management	Yes	101	90.2
	No	11	9.8
Intubation Technique *	Direct Laryngoscopy	74	73.3
	Fiberoptic Bronchoscopy	25	24.8
	Video Laryngoscopy	2	2.0
Tracheostomy	Yes	34	30.4
	No	78	69.6
Composite ICU Complication	Present	45	40.2
	Absent	67	59.8
Discharge Neurological Status	Favorable	47	42.0
	Unfavorable	65	58.0
Discharge Status (Three Categories) ^†^	Alive, ASIA D–E	47	42.0
	Alive, ASIA A–C	56	50.0
	In-Hospital Death	9	8.0
In-Hospital Mortality	Yes	9	8.0
	No	103	92.0

* Intubation technique is reported among the 101 patients who underwent operative management. ICU complications were recorded as a composite present/absent variable. The unfavorable discharge category includes nine in-hospital deaths; mortality is also shown separately. ^†^ Derived arithmetically from the counts above: unfavorable discharge status (*n* = 65) minus in-hospital deaths (*n* = 9).

**Table 3:** Exploratory Unadjusted Associations between Selected Clinical Variables and Discharge Neurological Status.

Variable	Category	Favorable *n* (%)	Unfavorable *n* (%)	*p*-Value	Unadjusted OR (95% CI)
Age	0–8 years	9 (56.2)	7 (43.8)	0.329	1.96 (0.67–5.72)
	9–18 years	38 (39.6)	58 (60.4)	Ref	1.00
Sex	Male	40 (43.0)	53 (57.0)	0.809	1.29 (0.47–3.58)
	Female	7 (36.8)	12 (63.2)	Ref	1.00
Mechanism of Injury	Fall	26 (39.4)	40 (60.6)	0.641	0.77 (0.36–1.66)
	RTA	21 (45.7)	25 (54.3)	Ref	1.00
Type of Spinal Injury	Fracture	35 (51.5)	33 (48.5)	0.024 *	2.74 (1.21–6.21)
	Subluxation	12 (27.9)	31 (72.1)	Ref	1.00
	SCIWORA	0 (0.0)	1 (100.0)	N/A ^‡^	—
Spinal Canal Compromise	Absent	19 (76.0)	6 (24.0)	<0.001 **	6.67 (2.40–18.54)
	Present	28 (32.2)	59 (67.8)	Ref	1.00
Tracheostomy	No	38 (48.7)	40 (51.3)	0.047 *	2.64 (1.09–6.37)
	Yes	9 (26.5)	25 (73.5)	Ref	1.00
Composite ICU Complication	Absent	39 (58.2)	28 (41.8)	<0.001 **	6.44 (2.61–15.93)
	Present	8 (17.8)	37 (82.2)	Ref	1.00
ASIA Category at Admission	Favorable (D–E)	24 (54.5)	20 (45.5)	0.048 *	2.35 (1.08–5.11)
	Unfavorable (A–C)	23 (33.8)	45 (66.2)	Ref	1.00

Chi-square test with Yates’ continuity correction (df = 1). * *p* < 0.05; ** *p* < 0.001. ^‡^ SCIWORA was excluded from the fracture-versus-subluxation comparison because *n* = 1. The unfavorable discharge category includes ASIA A–C and in-hospital death. OR = odds ratio; CI = confidence interval; RTA = road traffic accident; Ref = reference category. All ORs are unadjusted, are not corrected for multiple comparisons, and do not represent independent prognostic effects.

**Table 4:** Admission-to-Discharge Neurological Category Cross-Tabulation.

Admission ASIA Category	Discharge Favorable (D–E)	Discharge Unfavorable (A–C or Death)	Total
Favorable (D–E)	24	20	44
Unfavorable (A–C)	23	45	68
Total	47	65	112

The unfavorable discharge category includes ASIA A–C and in-hospital death. Individual A–E grade transitions and mortality stratified by admission ASIA grade were not reported in the original aggregate tables; the magnitude of grade-level change therefore cannot be determined from these data.

## Data Availability

All aggregate data supporting the analyses reported in this article are included in the manuscript and its tables. No additional dataset is available for secondary analysis.
